# Screening for diabetic peripheral neuropathy in resource-limited settings

**DOI:** 10.1186/s13098-023-01032-x

**Published:** 2023-03-22

**Authors:** Ken Munene Nkonge, Dennis Karani Nkonge, Teresa Njeri Nkonge

**Affiliations:** 1 University of Nairobi, 30197, Nairobi, Kenya; 2 McMaster University, Hamilton, ON L8S 4L8 Canada

**Keywords:** Diabetes, Diabetic foot syndrome, Diabetic peripheral neuropathy, Michigan neuropathy screening instrument, Neuropad, Risk factors, Screening, Treatment

## Abstract

**Background:**

Diabetic neuropathy is the most common microvascular complication of diabetes mellitus and a major risk factor for diabetes-related lower-extremity complications. Diffuse neuropathy is the most frequently encountered pattern of neurological dysfunction and presents clinically as distal symmetrical sensorimotor polyneuropathy. Due to the increasing public health significance of diabetes mellitus and its complications, screening for diabetic peripheral neuropathy is essential. Consequently, a review of the principles that guide screening practices, especially in resource-limited clinical settings, is urgently needed.

**Main body:**

Numerous evidence-based assessments are used to detect diabetic peripheral neuropathy. In accordance with current guideline recommendations from the American Diabetes Association, International Diabetes Federation, International Working Group on the Diabetic Foot, and National Institute for Health and Care Excellence, a screening algorithm for diabetic peripheral neuropathy based on multiphasic clinical assessment, stratification according to risk of developing diabetic foot syndrome, individualized treatment, and scheduled follow-up is suggested for use in resource-limited settings.

**Conclusions:**

Screening for diabetic peripheral neuropathy in resource-limited settings requires a practical and comprehensive approach in order to promptly identify affected individuals. The principles of screening for diabetic peripheral neuropathy are: multiphasic approach, risk stratification, individualized treatment, and scheduled follow-up. Regular screening for diabetes-related foot disease using simple clinical assessments may improve patient outcomes.

## Background

An unknown disease that caused excessive production of sweet urine was first described in historical records from Ancient Egypt, India, and China [1, 2]. Clinical experiences of numerous physicians and scientists including the Greek physician Aretaeus of Cappadocia (second century AD), the English anatomist and physician Thomas Willis (seventeenth century AD), and the English physician and physiologist Matthew Dobson (eighteenth century AD) subsequently contributed to widespread recognition of the disease, which is today known as diabetes mellitus (DM) [1].

DM has three microvascular complications: neuropathy, retinopathy, and nephropathy [3, 4]. Neuropathy can occur in patients with type 1 (T1DM) or type 2 DM (T2DM). The estimated prevalence of diabetic neuropathy among youth and adults is 2.4–75.1% according to data from cohort studies [5–7]. Diabetic neuropathy represents a clinically heterogeneous group of neurological disorders characterized by dysfunction of the peripheral nervous system attributed to DM after excluding other causes [8]. According to the pattern of neurological deficits, diabetic peripheral neuropathy can be painful or non-painful [9] and is classified into four types: diffuse neuropathy, mononeuropathy, radiculopathy, and other neuropathies [10]. Diffuse neuropathy is the most commonly encountered type and 75–90% of all diabetic peripheral neuropathy cases present with typical length-dependent sensorimotor symptoms characteristic of distal symmetrical polyneuropathy (DSPN) [10, 11]. Diabetic peripheral neuropathy is a major risk factor for lower-extremity complications such as ulceration, infection, deformity, and amputation. Research on diabetic peripheral neuropathy may help elucidate the complex pathogenetic mechanisms involved, thereby improving diagnosis and management [12].

Table 1 shows a chronology of important events in the history of diabetic peripheral neuropathy. Generally, the events can be grouped into two periods: foundation (second century AD—nineteenth century AD) and expansion (twentieth century AD—present).

**Table 1:** Milestones in the history of diabetic peripheral neuropathy

Year	Milestone	References
second century AD	Aretaeus of Cappadocia uses the word diabetes in his writings to describe a rare disease that causes excessive urination	[13]
1674	Thomas Willis uses the phrase, “quasi melle aut saccharo imbutam mire dulcescere” to describe the extremely sweet taste of urine from patients with diabetes and suggests that the sweetness is initially present in the blood	[14]
1776	Matthew Dobson conducts experiments that confirm the presence of sugar in urine and blood from patients with DM	[15]
1798	John Rollo provides detailed observations of symptoms consistent with dysfunction of the peripheral nervous system in patients with DM	[16]
1815	Michel Eugène Chevreul identifies glucose in urine from patients with DM	[14]
1864	Charles-Jacob Marchal de Calvi recognizes that DM causes dysfunction of the nervous system	[17]
1885	Frederick William Pavy describes the signs and symptoms of diabetic peripheral neuropathy	[18]
1887	Thomas Davies Pryce recognizes the relationship between peripheral nerve damage and foot ulceration in patients with DM	[19]
1887	Ernst Viktor von Leyden classifies diabetic peripheral neuropathy into three forms: hyperesthetic or neuralgic; motor or paralytic; and ataxic or pseudotabetic	[20]
1890	Jean-Martin Charcot describes the clinical features of diabetic peripheral neuropathy	[21]
1936	Harold Percival Himsworth recognizes that there are two main types of DM: insulin-sensitive or insulin-insensitive	[22]
1946–1947	The first community-based screening for DM is conducted in Oxford, Massachusetts	[23]
1954	M. Mencer Martin demonstrates the importance of neuropathy in the pathogenesis of foot lesions in patients with DM	[24]
1956	Wilfrid George Oakley classifies foot lesions in patients with DM into four types: septic; neuropathic; ischaemic; or combinations of septic, neuropathic, and ischaemic	[25]
1959	Sven-Erik Fagerberg recognizes that diabetic neuropathy is associated with histopathological changes in the small blood vessels of peripheral nerves	[26]
1961	Allan Watson Downie conducts research on nerve conduction velocities in patients with DM	[27]
1963	I. Steiness conducts research on vibration perception threshold in patients with DM	[28]
1988	A consensus panel proposes a scheme for classifying diabetic neuropathy into Class I (absence of demonstrable signs and symptoms) and Class II (presence of signs, symptoms, or both)	[29]
1988	Peter James Dyck proposes a system for staging the severity of diabetic neuropathy into grade 0 (no abnormality); grade 1a (nerve conduction abnormality); grade 1b (nerve conduction abnormality + signs); grade 2a (nerve conduction abnormality + symptoms ± signs; and grade 2b (nerve conduction abnormality + moderate weakness ± symptoms)	[30]
1994	The EDIC study commences with the aim of evaluating the development and progression of diabetes complications in the DCCT cohort	[31]
1997	Peter Kynaston Thomas proposes a scheme for classifying diabetic neuropathy into hyperglycemic neuropathy; symmetric polyneuropathy; focal and multifocal neuropathy; and mixed forms	[32]
1998	Andrew J. M. Boulton proposes a system for staging the severity of diabetic neuropathy into stage 0/1 (no clinical neuropathy); stage 2 (clinical neuropathy); stage 3 (late complications of clinical neuropathy)	[33]
2005	The ADA proposes a scheme for classifying diabetic neuropathy into two types: generalized symmetric polyneuropathies and focal and multifocal neuropathies	[34]
2008	Jennifer Tracy and Peter Dyck propose that diabetic neuropathy be classified either by anatomic pattern: symmetric and asymmetric or according to underlying pathophysiology: metabolic-microvascular-hypoxic; inflammatory immune; compression and repetitive injury; complications of diabetes; and treatment related	[35]
2010	The Toronto Diabetic Neuropathy Expert Group proposes diagnostic criteria for possible, probable, confirmed, and subclinical diabetic neuropathy	[36]
2017	The ADA proposes a comprehensive scheme for classifying diabetic neuropathy into diffuse neuropathy; mononeuropathy; and radiculopathy	[8]
twenty-first century AD	Development and implementation of comprehensive diabetic foot prevention programs gains momentum around the world	[37]

ADA: American Diabetes Association, DCCT: Diabetes Control and Complications Trial, DM: Diabetes mellitus, EDIC: Epidemiology of Diabetes Interventions and Complications

In the foundation period, major discoveries made by John Rollo in 1798, Charles-Jacob Marchal de Calvi in 1864, and Thomas Pryce in 1887 firmly established the relationship between DM, peripheral nerve dysfunction, and foot ulceration. Frederick Pavy described the signs and symptoms of diabetic peripheral neuropathy in 1885, while Ernst Viktor von Leyden proposed an early classification system that distinguished hyperesthetic, motor, and ataxic forms in 1887.

The expansion period is characterized by various advancements that are currently ongoing including: long-term population-based research on DM and its complications; use of objective approaches such as electrophysiological testing to detect nerve dysfunction in patients with diabetic peripheral neuropathy; refinement of diagnostic criteria and classification systems for diabetic peripheral neuropathy; and widespread implementation of structured foot screening programs for early detection of diabetic peripheral neuropathy and prevention of complications.

### Aims, material, and method

Neuropathy is an important risk factor for diabetes-related lower-extremity complications, which collectively contribute to the increasing global disability burden, especially among adults aged 50–69 years [38, 39]. Due to the public health implications of DM and its complications in resource-limited settings [40, 41], this narrative review aims to summarize the general principles of screening for diabetic peripheral neuropathy based on established and emerging evidence in order to delineate a practical approach to identifying adult patients at risk for diabetes-related foot disease and its complications.

Medical literature published in English between 1770 and 2023 was identified and considered for the review. The primary search strategy involved retrieval of relevant literature from health sciences databases (EMBASE, CINAHL, Cochrane library, and PubMed) and grey literature (Google Scholar, Opengrey, Scopus, Virtual Health Library, Web of Science Core Collection, and organization websites) using a combination of keywords and Boolean operators: “comprehensive foot examination” AND “diabetic foot”; diabetes AND “microvascular complications”; (“diabetic foot” OR “diabetic foot syndrome”); (“diabetic peripheral neuropathy” OR “distal symmetrical polyneuropathy”); “diabetic peripheral neuropathy” AND guideline; epidemiology AND “diabetic peripheral neuropathy”; (“non-painful diabetic neuropathy” OR “painless diabetic neuropathy”); “painful diabetic neuropathy” AND treatment; “diabetic peripheral neuropathy” AND “risk factors”; prevention AND “diabetic peripheral neuropathy”; and Screening AND “diabetic peripheral neuropathy”. The secondary search strategy involved citation searching in order to retrieve additional relevant literature.

Epidemiological data, screening practices, and management strategies were extracted from the retrieved literature. Comprehensive findings were summarized and reported qualitatively.

## Findings

### Epidemiology of diabetic peripheral neuropathy

Epidemiological data for diabetic peripheral neuropathy is heterogeneous. The reasons for heterogeneity include: large proportion of asymptomatic patients [8]; few population-based studies reported in the literature [42–46]; differences in the burden of neuropathy in patients with T1DM compared to T2DM [47–49]; limited research on painful and non-painful variants of neuropathy [9, 50, 51]; and lack of a standardized approach to screening.

The prevalence of diabetic peripheral neuropathy is known to increase with age and is estimated to be 6–60% among adult patients [47, 52]. Painful diabetic neuropathy (PDN) is particularly common in adults and has an estimated prevalence of 10–68% among diverse patient cohorts [53–56]. Therefore, screening for diabetic peripheral neuropathy is an important preventive care practice that may lead to a substantial reduction in disease burden.

### Screening for diabetic peripheral neuropathy

#### Principle 1: multiphasic approach

It is beyond the scope of the present article to discuss the various methods used to detect diabetic peripheral neuropathy. The methods have been comprehensively reviewed in various publications [57–61].

Current screening practices are region specific, but the position statement by the American Diabetes Association (ADA) and the ADA evidence-based standards of care in diabetes guideline provide comprehensive guidance [8, 62]. The ADA recommends that medical history and comprehensive foot examination be used to screen for diabetic peripheral neuropathy at time of diagnosis for patients with T2DM and five years after diagnosis for patients with T1DM. Furthermore, patients should be reassessed at least annually regardless of DM type using 10-g Semmes–Weinstein monofilament evaluation (SWME) and at least one other clinical assessment such as vibration perception, pinprick, temperature perception, or ankle reflexes [62].

The ADA does not recommend assessment of sudomotor function during clinical evaluation of diabetic peripheral neuropathy [62]. Instead, pinprick and temperature sensation are recommended for assessment of small nerve fiber function. However, sudomotor dysfunction is a critical pathophysiological process in the pathogenesis of diabetes-related foot disease, especially in the early stages [63–65]. Research suggests that evaluation of sudomotor function helps identify individuals at risk for foot ulceration [66, 67], but whether such evaluation should be conducted routinely is unclear. Neuropad is an accurate, sensitive, and cost-effective point-of-care test that is used to evaluate sudomotor function [65, 68–71]. Since Neuropad has high sensitivity and negative predictive value for detecting small nerve fiber dysfunction [72, 73], it may be used as an adjunct clinical test during diabetic foot screening. However, further validation of Neuropad is needed to support its widespread use during foot screening.

Lack of a standardized methodology for the screening of DM or its microvascular complications in resource-limited settings is an unmet medical need [74]. Additionally, there is no single tool that can be used to objectively evaluate sensory, motor, and autonomic deficits associated with diabetic peripheral neuropathy [58]. Evidence suggests that combining multiple assessments increases sensitivity, specificity, and accuracy of detecting diabetic peripheral neuropathy [8, 75–78]. Therefore, multiphasic screening—where one or more tools for assessing the signs and symptoms of diabetic peripheral neuropathy are used concurrently or sequentially—may detect a greater proportion of deficits, thereby aiding clinical decision-making. Through the multiphasic approach, both small and large nerve fiber function can be evaluated using objective measures of diabetic peripheral neuropathy [79]. In resource-limited settings, a practical combination of assessments could be focused medical history, Michigan Neuropathy Screening Instrument (MNSI), and a simple point-of-care test such as Neuropad.

The MNSI is comprised of a questionnaire (MNSIQ) and physical examination (MNSIE). Both components are sensitive, specific, and easy to administer [80, 81]. MNSIQ scores  ≥ 4 and MNSIE scores  ≥ 2 are abnormal and suggest diabetic peripheral neuropathy in patients with T1DM [82] while MNSIQ  ≥ 7 and MNSIE  ≥ 2 are suggestive of diabetic peripheral neuropathy in patients with T2DM [83].

The Neuropad test involves placing a blue plaster impregnated with anhydrous cobalt-II-chloride on the plantar aspect of the foot and observing for a change in color over 10–15 min [84–86]. If the plaster remains blue or turns patchy blue/pink there is inadequate sweat production due to sudomotor dysfunction, which indicates increased risk for foot ulceration [84–87].

Recently, the combinations of MNSI and SUDOSCAN [88, 89] or MNSI, Neuropad, and Vibratip have been used to rapidly and non-invasively detect diabetic peripheral neuropathy [90]. Overall, evidence from such studies and current guideline recommendations support the use of various combinations of assessments during screening for diabetic peripheral neuropathy.

#### Principle 2: risk stratification

The second objective of screening is prevention of diabetic foot syndrome (DFS), which is a complication of diabetic peripheral neuropathy associated with high rates of hospitalization and non-traumatic lower-extremity amputation [91, 92]. DFS is a pathological condition characterized by ulceration, infection, or deformity of the foot due to diabetes-related neurovascular dysfunction. Over 80% of lower-extremity amputations in patients with DM are preceded by foot ulceration [93, 94]. Four pathophysiological processes are implicated in diabetic foot ulceration (DFU): loss of protective sensation (LOPS) secondary to DSPN, ischemia secondary to peripheral arterial disease (PAD), anhidrosis and arteriovenous shunting secondary to peripheral autonomic neuropathy, and repetitive trauma [91]. The lifetime risk of developing DFU is between 19 and 34% [95, 96]. Rates of ulcer recurrence after healing are estimated to be 40% within one year, 60% within three years, and 65% within five years [95]. The corresponding one-, three-, and five-year survival rates associated with DFU are 86.9%, 66.9%, and 50.9%, respectively [97]. The highest risk of mortality related to DFU is reported for patients with end-stage renal disease (ESRD), amputation, chronic kidney disease, PAD, older age, or a history of cardiovascular disease [97]. Despite the public health significance of DFS, very little is known about its true burden in resource-limited settings [98, 99].

Based on current guidelines, there are several criteria for stratifying patients according to their clinical risk for DFS and amputation [100–103]. The criteria are summarized below.

The International Diabetes Federation (IDF) stratifies patients into four groups:

- Low risk—normal plantar sensation
- Moderate risk—presence of LOPS
- High risk—presence of LOPS ± PAD
- Very high risk—history of ulceration, amputation, or neuropathic fracture

The International Working Group on the Diabetic Foot (IWGDF) stratifies patients into four categories:

- Very low risk—absence of LOPS or PAD
- Low risk— presence of LOPS or PAD
- Moderate risk— presence of LOPS + PAD; or LOPS + deformity; or PAD + deformity
- High risk—presence of LOPS or PAD + one or more of the following: previous foot ulceration; or lower-extremity amputation (major or minor); or ESRD

The ADA stratifies patients into five categories:

- Very low risk—absence of LOPS and PAD
- Low risk—presence of LOPS ± deformity
- Moderate risk—presence of PAD ± LOPS; diminished dorsalis pedis or posterior tibial pulse; presence of swelling or edema
- High risk—presence of DM with previous history of ulceration or lower-extremity amputation; chronic venous insufficiency
- Urgent—active foot pathology: open wound or ulcerative area ± signs of infection; new neuropathic pain or pain at rest; signs of active Charcot deformity; vascular compromise

The National Institute for Health and Care Excellence (NICE) stratifies patients into four groups:

- Low risk—presence of callus alone
- Moderate risk—presence of deformity; or neuropathy; or PAD
- High risk—previous ulceration; or previous amputation; or on renal replacement therapy; or neuropathy + PAD; or neuropathy + callus ± deformity; or PAD + callus ± deformity
- Active diabetic foot—presence of ulceration; or foot infection; or chronic limb-threatening ischaemia; or gangrene; or suspicion of acute Charcot arthropathy

Patients at increased risk for DFS and lower-extremity amputation are recommended to undergo SWME, despite concerns about diagnostic accuracy of SWME during screening for diabetes-related foot disease [104–107] and questions about the number of sites on the foot that must be assessed [106, 107]. In settings where SWME cannot be conducted, the Ipswich Touch Test (IpTT) is a potential substitute assessment that may be used to evaluate LOPS [108–110]. However, since no clinical guidelines currently recommend IpTT for risk stratification, more studies are needed to evaluate whether it is an appropriate substitute for SWME.

A recently developed semi-quantitative scoring system stratifies patients according to their risk for developing DFU based on minor criteria (foot or nail fungal infection; ill-fitting socks and footwear; lack of visual or cognitive ability for selfcare; glycated hemoglobin  > 9%; diabetes duration  > 10 years; and male sex), moderate criteria (slight polyneuropathy; pronounced foot deformity; pronounced hyperkeratosis; PAD; and renal insufficiency or dialysis), and major criteria (pronounced polyneuropathy; history of foot ulcer; and history of non-traumatic amputation) [111]. Validation of this new risk assessment system in diverse patients at risk for DFS is needed to determine how to incorporate the system into future screening practices.

#### Principle 3: individualized treatment and scheduled follow-up

Management of diabetic peripheral neuropathy remains a challenge for health care providers because none of the currently available treatments effectively target underlying pathogenesis [112]. Furthermore, diabetic peripheral neuropathy is a progressive disorder that can cause irreversible nerve damage [113, 114]. Thus, individualized treatment and scheduled follow-up are interdependent. The main objectives of treatment are intensive glycemic control and management of neuropathic pain alongside one or all of the following: diabetes self-management education and support; lifestyle optimization; adequate foot care and proper or therapeutic footwear; and multifactorial control of cardiovascular risk factors [112, 115–117].

PDN is particularly difficult to manage and current treatment options include pharmacologic and non-pharmacologic interventions [117, 118]. Four classes of oral pharmacologic interventions are recommended for treatment: gabapentinoids (gabapentin, mirogabalin, and pregabalin); serotonin-norepinephrine reuptake inhibitors (desvenlafaxine, duloxetine, and venlafaxine); tricyclic antidepressants (amitriptyline, imipramine, and nortriptyline); and sodium channel blockers (carbamazepine, lacosamide, lamotrigine, oxcarbazepine, and valproic acid) [118]. Topical treatment with capsaicin may be considered in patients with contraindications to oral pharmacotherapy or a preference for topical pain management [118]. Due to their adverse events profile and high abuse potential, serotonin-norepinephrine reuptake inhibitors/opioid dual mechanism agents (tapentadol and tramadol) are currently not recommended for treating PDN [118]. Unfortunately, pharmacologic intervention seldom achieves complete resolution of neuropathic pain due to limited efficacy, dose-limiting adverse events, or both [119–121]. Therefore, a standardized approach to combining pharmacotherapies is an unmet medical need and remains an area of intense study [122–124].

Spinal cord stimulation is an emerging therapeutic adjunct for the management of PDN [125]. This type of non-pharmacologic intervention—referred to as neuromodulation—effectively relieves pain, improves neurological function, and enhances quality of life [126, 127]. High-quality evidence supports the use of either invasive or non-invasive neuromodulation for the treatment of patients with PDN that is refractory to pharmacologic intervention [128, 129].

Finally, health care providers should remember that patients with asymmetrical distribution of clinical signs and symptoms or an unclear diagnosis require prompt referral to a neurologist for confirmatory electrophysiological testing [8].

### Integrated approach to screening for diabetes-related foot disease

**Fig. 1:**
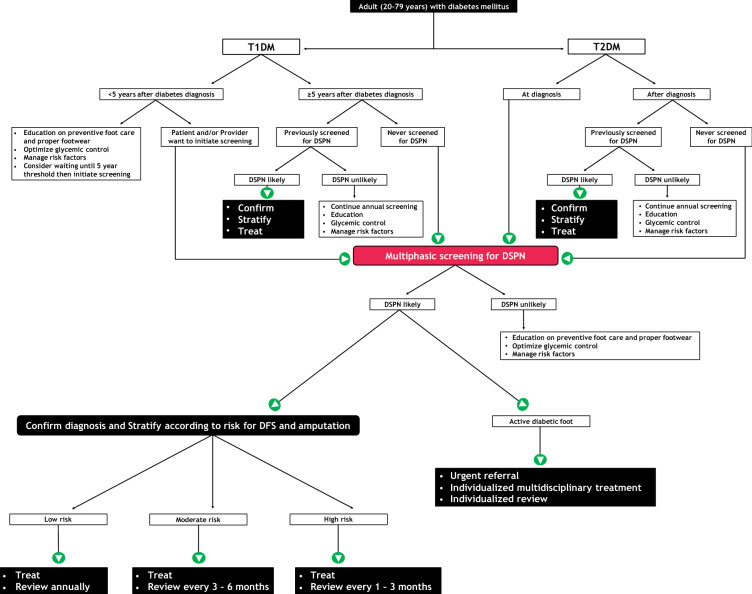
Proposed screening algorithm for diabetic peripheral neuropathy. Screening for diabetic peripheral neuropathy involves multiphasic clinical assessment, stratification according to risk for diabetic foot syndrome and amputation, and individualized treatment and scheduled follow-up. DFS: diabetic foot syndrome; DSPN: distal symmetrical polyneuropathy; T1DM: type 1 diabetes mellitus; T2DM: type 2 diabetes mellitus

Figure 1 shows a comprehensive algorithm that integrates the general principles of screening for diabetic peripheral neuropathy. This algorithm may assist clinical decision-making in resource-limited settings and contribute to standardization of preventive foot screening practices.

In Sheffield, United Kingdom, a one-stop microvascular complication screening clinic has used a similar algorithm/flow chart to screen patients for diabetic peripheral neuropathy as part of its comprehensive diabetes-related foot, eye, and renal disease assessment [130]. This shift towards integrated screening programs is a potentially transformative development because health care providers may be able to detect individuals at risk or those already affected more efficiently and utilize available healthcare resources more effectively [130–132].

Evidence from available studies suggests that concurrent screening for one or more diabetes-related complications is well-received by patients and health care providers and may be more convenient than attending separate screening sessions [133, 134]. The current limitations of clinical guidelines and unique challenges affecting diverse clinical settings must be overcome in order to optimize preventive foot screening practices and facilitate implementation of concurrent screening strategies for microvascular complications of DM [135–137].

## Conclusions

In conclusion, diabetic peripheral neuropathy represents a clinically heterogeneous group of neurological disorders that are classified according to the pattern of neurological dysfunction. Diabetic peripheral neuropathy is highly prevalent and associated with substantial morbidity and mortality. Regular screening for diabetes-related foot disease using simple assessments may improve patient outcomes. Multiphasic clinical assessment for diabetic peripheral neuropathy, risk stratification, individualized treatment, and scheduled follow-up of patients is a practical approach to preventive foot care in resource-limited settings.

Areas of ongoing research that are expected to have a positive impact on future screening practice include: identification of a single tool that accurately detects sensory, motor, and autonomic nerve dysfunction in diabetic peripheral neuropathy; elucidation of the prevalence of diabetic peripheral neuropathy in diverse patient populations; validation of recently proposed systems for risk stratifying patients with diabetes-related foot disease; and discovery of novel pharmacologic and non-pharmacologic treatments for diabetic peripheral neuropathy.

## Data Availability

Not applicable.

## Abbreviations

ADA: American Diabetes Association
DCCT: Diabetes control and complications trial
DFS: Diabetic foot syndrome
DFU: Diabetic foot ulceration
DM: Diabetes mellitus
DSPN: Distal symmetrical polyneuropathy
EDIC: Epidemiology of diabetes interventions and complications
ESRD: End-stage renal disease
IDF: International diabetes federation
IpTT: Ipswich touch test
IWGDF: International working group on the diabetic foot
LOPS: Loss of protective sensation
MNSI: Michigan neuropathy screening instrument
MNSIE: Michigan neuropathy screening instrument examination
MNSIQ: Michigan neuropathy screening instrument questionnaire
NICE: National institute for health and care excellence
PAD: Peripheral arterial disease
PDN: Painful diabetic neuropathy
SWME: Semmes–weinstein monofilament evaluation
T1DM: Type 1 diabetes mellitus
T2DM: Type 2 diabetes mellitus

## References

[1] Karamanou M, Protogerou A, Tsoucalas G, Androutsos G, Poulakou-Rebelakou E (2016). Milestones in the history of diabetes mellitus: the main contributor. World J Diabetes.

[2] Gwei-Djen L, Needham J (1976). Records of disease in ancient China. Am J Chin Med.

[3] Nathan DM (1993). Long-term complications of diabetes mellitus. N Engl J Med.

[4] Tommerdahl KL, Shapiro ALB, Nehus EJ, Bjornstad P (2022). Early microvascular complications in type 1 and type 2 diabetes: recent developments and updates. Pediatr Nephrol.

[5] Kostev K, Jockwig A, Hallwachs A, Rathmann W (2014). Prevalence and risk factors of neuropathy in newly diagnosed type 2 diabetes in primary care practices: a retrospective database analysis in Germany and U.K. Prim Care Diabetes.

[6] Jaiswal M, Divers J, Dabelea D, Isom S, Bell RA, Martin CL, Pettitt DJ, Saydah S, Pihoker C, Standiford DA, Dolan LM, Marcovina S, Linder B, Liese AD, Pop-Busui R, Feldman EL (2017). Prevalence of and risk factors for diabetic peripheral neuropathy in youth with type 1 and type 2 diabetes: SEARCH for diabetes in youth study. Diabetes Care.

[7] Janghorbani M, Rezvanian H, Kachooei A, Ghorbani A, Chitsaz A, Izadi F, Amini M (2006). Peripheral neuropathy in type 2 diabetes mellitus in Isfahan, Iran: prevalence and risk factors. Acta Neurol Scand.

[8] Pop-Busui R, Boulton AJM, Feldman EL, Bril V, Freeman R, Malik RA (2017). Diabetic neuropathy: a position statement by the American diabetes association. Diabetes Care.

[9] Jensen TS, Karlsson P, Gylfadottir SS, Andersen ST, Bennett DL, Tankisi H (2021). Painful and non-painful diabetic neuropathy, diagnostic challenges and implications for future management. Brain.

[10] Ziegler D, Papanas N, Schnell O, Nguyen BDT, Nguyen KT, Kulkantrakorn K, Deerochanawong C (2021). Current concepts in the management of diabetic polyneuropathy. J Diabetes Investig.

[11] Kasznicki J (2014). Advances in the diagnosis and management of diabetic distal symmetric polyneuropathy. Arch Med Sci.

[12] Aso Y (2022). Updates in diabetic neuropathy: a call for new diagnostic and treatment approaches. J Diabetes Investig.

[13] Laios K, Karamanou M, Saridaki Z, Androutsos G (2012). Aretaeus of Cappadocia and the first description of diabetes. Hormones.

[14] Eknoyan G, Nagy J (2005). A history of diabetes mellitus or how a disease of the kidneys evolved into a kidney disease. Adv Chronic Kidney Dis.

[15] Dobson M. 1776 Experiments and observations on the urine in a diabetes. Med Obs Inq. 5:298–316

[16] Rollo J. Cases of the diabetes mellitus with the results of the trials of certain acids, in the cure of the lues venerea. 2nd ed. London: T. Gillet, for C. Dilly, in the Poultry; 1798.

[17] de Calvi CJM. Recherches sur les accidents diabétiques et essai d’une théorie générale du diabète. Forgotten Books. 2019.

[18] Pavy FW (1885). Introductory address to the discussion on the clinical aspects of glycosuria. Lancet.

[19] Pryce TD (1887). A case of perforating ulcers of both feet associated with diabetes and ataxic symptoms. Lancet.

[20] Leyden E. Die entzündung der peripheren nerven. Deut Militäräztl Zeitschr.1888.

[21] Charcot JM (1890). Sur un cas de paraplégie diabétique. Arch Neurol.

[22] Himsworth HP (1936). Diabetes mellitus: its differentiation into insulin-sensitive and insulin-insensitive types. Lancet.

[23] Wilkerson HLC, Krall LP (1947). Diabetes in a New England town; a study of 3,516 persons in Oxford. Mass J Am Med Assoc.

[24] Martin MM (1954). Neuropathic lesions of the feet in diabetes mellitus. Proc R Soc Med.

[25] Oakley W, Catterall RCF, Martin MM (1956). Aetiology and management of lesions of the feet in diabetes. Br Med J.

[26] Fagerberg SE (1959). Diabetic neuropathy: a clinical and histological study on the significance of vascular affections. Acta Med Scand Suppl.

[27] Downie AW, Newell DJ (1961). Sensory nerve conduction in patients with diabetes mellitus and controls. Neurology.

[28] Steiness I (1963). Diabetic neuropathy. Vibration sense and abnormal reflexes in diabetics. Acta Med Scand Suppl.

[29] Consensus statement: report and recommendations of the San Antonio conference on diabetic neuropathy. Diabetes Care. 1988;11(7):592–7. 10.2337/diacare.11.7.592. 10.2337/diacare.11.7.592 3060328

[30] Dyck PJ (1988). Detection, characterization, and staging of polyneuropathy: assessed on diabetics. Muscle Nerve.

[31] Epidemiology of Diabetes Interventions and Complications (EDIC) Research Group (1999). Epidemiology of diabetes interventions and complications (EDIC) Design, implementation, and preliminary results of a long-term follow-up of the diabetes control and complications trial cohort. Diabetes Care.

[32] Thomas PK (1997). Classification, differential diagnosis, and staging of diabetic peripheral neuropathy. Diabetes.

[33] Boulton AJ, Gries FA, Jervell JA (1998). Guidelines for the diagnosis and outpatient management of diabetic peripheral neuropathy. Diabet Med.

[34] Boulton AJM, Vinik AI, Arezzo JC, Bril V, Feldman EL, Freeman R (2005). Diabetic neuropathies: a statement by the American diabetes association. Diabetes Care.

[35] Tracy JA, Dyck PJB (2008). The spectrum of diabetic neuropathies. Phys Med Rehabil Clin N Am.

[36] Tesfaye S, Boulton AJM, Dyck PJ, Freeman R, Horowitz M, Kempler P (2010). Diabetic neuropathies: update on definitions, diagnostic criteria, estimation of severity, and treatments. Diabetes Care.

[37] Lavery LA, Oz OK, Bhavan K, Wukich DK (2019). Diabetic foot syndrome in the twenty-first century. Clin Podiatr Med Surg.

[38] Lazzarini PA, Pacella RE, Armstrong DG, van Netten JJ (2018). Diabetes-related lower-extremity complications are a leading cause of the global burden of disability. Diabet Med.

[39] Zhang Y, Lazzarini PA, McPhail SM, van Netten JJ, Armstrong DG, Pacella RE (2020). Global disability burdens of diabetes-related lower-extremity complications in 1990 and 2016. Diabetes Care.

[40] Dunachie S, Chamnan P (2019). The double burden of diabetes and global infection in low and middle-income countries. Trans R Soc Trop Med Hyg.

[41] Godman B, Basu D, Pillay Y, Mwita JC, Rwegerera GM, Anand Paramadhas BD, Tiroyakgosi C (2020). Review of ongoing activities and challenges to improve the care of patients with type 2 diabetes across Africa and the implications for the future. Front Pharmacol.

[42] Abbott CA, Malik RA, van Ross ERE, Kulkarni J, Boulton AJM (2011). Prevalence and characteristics of painful diabetic neuropathy in a large community-based diabetic population in the U.K. Diabetes Care.

[43] Kärvestedt L, Mårtensson E, Grill V, Elofsson S, von Wendt G, Hamsten A, Brismar K (2011). The prevalence of peripheral neuropathy in a population-based study of patients with type 2 diabetes in Sweden. J Diabetes Complications.

[44] Katulanda P, Ranasinghe P, Jayawardena R, Constantine GR, Sheriff MHR, Matthews DR (2012). The prevalence, patterns and predictors of diabetic peripheral neuropathy in a developing country. Diabetol Metab Syndr.

[45] Khedr EM, Fawi G, Allah Abbas MA, El-Fetoh NA, Al Attar G, Zaki AF, Gamea A (2016). Prevalence of diabetes and diabetic neuropathy in Qena Governorate: population-based survey. Neuroepidemiology.

[46] Yovera-Aldana M, Velásquez-Rimachi V, Huerta-Rosario A, More-Yupanqui MD, Osores-Flores M, Espinoza R (2021). Prevalence and incidence of diabetic peripheral neuropathy in Latin America and Caribbean: a systematic review and meta-analysis. PLoS ONE.

[47] Pfannkuche A, Alhajjar A, Ming A, Walter I, Piehler C, Mertens PR (2020). Prevalence and risk factors of diabetic peripheral neuropathy in a diabetics cohort: register initiative “diabetes and nerves”. Endocr Metab Sci.

[48] Galosi E, Hu X, Michael N, Nyengaard JR, Truini A, Karlsson P (2022). Redefining distal symmetrical polyneuropathy features in type 1 diabetes: a systematic review. Acta Diabetol.

[49] Akinci G, Savelieff MG, Gallagher G, Callaghan BC, Feldman EL (2021). Diabetic neuropathy in children and youth: new and emerging risk factors. Pediatr Diabetes.

[50] Barrett AM, Lucero MA, Le T, Robinson RL, Dworkin RH, Chappell AS (2007). Epidemiology, public health burden, and treatment of diabetic peripheral neuropathic pain: a review. Pain Med.

[51] Themistocleous AC, Ramirez JD, Shillo PR, Lees JG, Selvarajah D, Orengo C (2016). The pain in neuropathy study (PiNS): a cross-sectional observational study determining the somatosensory phenotype of painful and painless diabetic neuropathy. Pain.

[52] Hicks CW, Selvin E (2019). Epidemiology od peripheral neuropathy and lower extremity disease in diabetes. Curr Diab Rep.

[53] Gylfadottir SS, Christensen DH, Nicolaisen SK, Andersen H, Callaghan BC, Itani M (2020). Diabetic polyneuropathy and pain, prevalence, and patient characteristics: a cross-sectional questionnaire study of 5514 patients with recently diagnosed type 2 diabetes. Pain.

[54] Veves A, Backonja M, Malik RA (2008). Painful diabetic neuropathy: epidemiology, natural history, early diagnosis, and treatment options. Pain Med.

[55] Halawa MR, Karawagh A, Zeidan A, Mahmoud AE, Sakr M, Hegazy A (2010). Prevalence of painful diabetic peripheral neuropathy among patients suffering from diabetes mellitus in Saudi Arabia. Curr Med Res Opin.

[56] Mizokami-Stout KR, Li Z, Foster NC, Shah V, Aleppo G, McGill JB, Pratley K, Toschi E, Ang L, Pop-Busui R, for T1D Exchange Clinic Network (2020). The contemporary prevalence of diabetic neuropathy in type 1 diabetes: findings from the T1D Exchange. Diabetes Care.

[57] Bönhof GJ, Herder C, Strom A, Papanas N, Roden M, Ziegler D (2019). Emerging biomarkers, tools, and treatments for diabetic polyneuropathy. Endocr Rev.

[58] Carmichael J, Fadavi H, Ishibashi F, Shore AC, Tavakoli M (2021). Advances in screening, early diagnosis and accurate staging of diabetic neuropathy. Front Endocrinol.

[59] Won JC, Park TS (2016). Recent advances in diagnostic strategies for diabetic peripheral neuropathy. Endocrinol Metab.

[60] Yu Y (2021). Gold standard for diagnosis of DPN. Front Endocrinol.

[61] Newlin Lew K, Arnold T, Cantelmo C, Jacque F, Posada-Quintero H, Luthra P, Chon KH (2022). Diabetes distal peripheral neuropathy: subtypes and diagnostic and screening technologies. J Diabetes Sci Technol.

[62] ElSayed NA, Aleppo G, Aroda VR, Bannuru RR, Brown FM, Bruemmer D, Collins BS, Gibbons CH, Giurini JM, Hilliard ME, Isaacs D, Johnson EL, Kahan S, Khunti K, Leon J, Lyons SK, Perry ML, Prahalad P, Pratley RE, Seley JJ, Stanton RC, Sun JK, Gabbay RA, on behalf of the American Diabetes Association (2023). Retinopathy, neuropathy, and foot care: standards of medical care in diabetes-2023. Diabetes Care.

[63] Boulton AJ, Malik RA (1998). Diabetic neuropathy. Med Clin North Am.

[64] Mascarenhas JV, Jude EB (2014). The Charcot foot as a complication of diabetic neuropathy. Curr Diab Rep.

[65] Sharma S, Vas P, Rayman G (2022). Small fiber neuropathy in diabetes polyneuropathy: is it time to change?. J Diabetes Sci Technol.

[66] Tentolouris N, Marinou K, Kokotis P, Karanti A, Diakoumopoulou E, Katsilambros N (2009). Sudomotor dysfunction is associated with foot ulceration in diabetes. Diabet Med.

[67] Yajnik CS, Kantikar VV, Pande AJ, Deslypere JP (2012). Quick and simple evaluation of sudomotor function for screening of diabetic neuropathy. ISRN Endocrinol.

[68] Papanas N, Paschos P, Papazoglou D, Papatheodorou K, Paletas K, Maltezos E (2011). Accuracy of the Neuropad test for the diagnosis of distal symmetric polyneuropathy in type 2 diabetes. Diabetes Care.

[69] Quattrini C, Jeziorska M, Tavakoli M, Begum P, Boulton AJM, Malik RA (2008). The Neuropad test: a visual indicator test for human diabetic neuropathy. Diabetologia.

[70] Tsapas A, Laikos A, Paschos P, Karagiannis T, Bekiari E, Tentolouris N, Boura P (2014). A simple plaster for screening for diabetic neuropathy: a diagnostic test accuracy systematic review and meta-analysis. Metabolism.

[71] Rodríguez-Sánchez B, Peña-Longobardo LM, Sinclair AJ (2020). Cost-effectiveness analysis of the Neuropad device as a screening tool for early diabetic peripheral neuropathy. Eur J Health Econ.

[72] Ponirakis G, Petropoulos IN, Fadavi H, Alam U, Asghar O, Marshall A (2014). The diagnostic accuracy of Neuropad for assessing large and small fibre diabetic neuropathy. Diabet Med.

[73] Manes C, Papanas N, Exiara T, Katsiki N, Papantoniou S, Kirlaki E (2014). The indicator test Neuropad in the assessment of small and overall nerve fibre dysfunction in patients with type 2 diabetes: a large multicentre study. Exp Clin Endocrinol Diabetes.

[74] Grant P (2013). Management of diabetes in resource-poor settings. Clin Med.

[75] Armstrong DG, Lavery LA, Vela SA, Quebedeaux TL, Fleischli JG (1998). Choosing a practical screening instrument to identify patients at risk for diabetic foot ulceration. Arch Intern Med.

[76] Al-Geffari M (2012). Comparison of different screening tests for diagnosis of diabetic peripheral neuropathy in primary health care setting. Int J Health Sci.

[77] Jie FY, Zafar MI, Xu L, Shafqat RA, Gao F (2018). Sensitivity of four simple methods to screen Chinese patients for diabetic peripheral neuropathy. Acta Endocrinol.

[78] Ang L, Cowdin N, Mizokami-Stout K, Pop-Busui R (2018). Update on the management of diabetic neuropathy. Diabetes Spectr.

[79] Fernández-Torres R, Ruiz-Muñoz M, Pérez-Panero AJ, García-Romero J, Gónzalez-Sánchez M (2020). Instruments of choice for assessment and monitoring diabetic foot: a systematic review. J Clin Med.

[80] Feldman EL, Stevens MJ (1994). Clinical testing in diabetic peripheral neuropathy. Can J Neurol Sci.

[81] Moghtaderi A, Bakhshipor A, Rashidi H (2006). Validation of Michigan neuropathy screening instrument for diabetic peripheral neuropathy. Clin Neurol Neurosurg.

[82] Herman WH, Pop-Busui R, Braffett BH, Martin CL, Cleary PA, Albers JW (2012). Use of the Michigan neuropathy screening instrument as a measure of distal symmetrical peripheral neuropathy in type 1 diabetes: results from the diabetes control and complications trial/epidemiology of diabetes interventions and complications. Diabet Med.

[83] Yeboah K, Puplampu P, Boima V, Antwi DA, Gyan B, Amoah AGB (2016). Peripheral sensory neuropathy in type 2 diabetes patients: a case control study in Accra. Ghana J Clin Transl Endocrinol.

[84] Papanas N, Boulton AJM, Malik RA, Manes C, Schnell O, Spallone V (2013). A simple new non-invasive sweat indicator test for the diagnosis of diabetic neuropathy. Diabet Med.

[85] Spallone V, Morganti R, Siampli M, Fedele T, D’Amato C, Cacciotti L, Maiello MR (2009). Neuropad as a diagnostic tool for diabetic autonomic and sensorimotor neuropathy. Diabet Med.

[86] Ziegler D, Papanas N, Roden M, GDC Study Group (2011). Neuropad: evaluation of three cut-off points of sudomotor dysfunction for early detection of polyneuropathy in recently diagnosed diabetes. Diabet Med.

[87] Panagoulias GS, Eleftheriadou I, Papanas N, Manes C, Kamenov Z, Tesic D (2020). Dryness of foot skin assessed by the visual indicator test and risk of diabetic foot ulceration: a prospective observational study. Front Endocrinol.

[88] Oh TJ, Song Y, Jang HC, Choi SH (2021). SUDOSCAN in combination with the Michigan neuropathy screening instrument is an effective tool for screening diabetic peripheral neuropathy. Diabetes Metab J.

[89] Carbajal-Ramírez A, Hernández-Domínguez JA, Molina-Ayala MA, Rojas-Uribe MM, Chávez-Negrete A (2019). Early identification of peripheral neuropathy based on sudomotor dysfunction in Mexican patients with type 2 diabetes. BMC Neurol.

[90] Gómez-Banoy N, Cuevas V, Soler F, Pineda MF, Mockus I (2017). Screening tests for distal symmetrical polyneuropathy in Latin American patients with type 2 diabetes mellitus. Arch Endocrinol Metab.

[91] Miranda C, Da Ros R, Marfella R (2021). Update on prevention of diabetic foot ulcer. Arch Med Sci Atheroscler Dis.

[92] Volmer-Thole M, Lobmann R (2016). Neuropathy and diabetic foot syndrome. Int J Mol Sci.

[93] Pecoraro RE, Reiber GE, Burgess EM (1990). Pathways to diabetic limb amputation basis for prevention. Diabetes Care.

[94] Reiber GE (1996). The epidemiology of diabetic foot problems. Diabet Med.

[95] Armstrong DG, Boulton AJM, Bus SA (2017). Diabetic foot ulcers and their recurrence. N Engl J Med.

[96] Edmonds M, Manu C, Vas P (2021). The current burden of diabetic foot disease. J Clin Orthop Trauma.

[97] Chen L, Sun S, Gao Y, Ran X (2023). Global mortality of diabetic foot ulcer: a systematic review and meta-analysis of observational studies. Diabetes Obes Metab.

[98] Abbas ZG (2017). Managing the diabetic foot in resource-poor settings: challenges and solutions. Chronic Wound Care Manag Res.

[99] Abbas ZG, Boulton AJM (2021). Diabetic foot ulcer disease in African continent: ‘from clinical care to implementation’—review of diabetic foot in last 60 years – 1960 to 2020. Diabetes Res Clin Pract.

[100] Ibrahim A, Jude E, Langton K, Jesus FRM, Harkless LB, Gawish H, et al. IDF Clinical practice recommendations on the diabetic foot—2017: a guide for health care professionals. Belgium: International Diabetes Federation; 2017. https://www.idf.org/e-library/guidelines/119-idf-clinical-practice-recommendations-on-diabetic-foot-2017.html. Accessed 15 Dec 2021.

[101] Schaper NC, van Netten JJ, Apelqvist J, Bus SA, Hinchliffe RJ, Lipsky BA, IWGDF Editorial Board (2020). Practical guidelines on the prevention and management of diabetic foot disease (IWGDF 2019 update). Diabetes Metab Res Rev.

[102] Boulton AJM, Armstrong DG, Kirsner RS, Attinger CE, Lavery LA, Lipsky BA, et al. Diagnosis and management of diabetic foot complications. Arlington (VA): American diabetes association; 2018. https://www.ncbi.nlm.nih.gov/books/NBK538977/. Accessed 15 Dec 2021. 30958663

[103] National Institute for Health and Care Excellence. Diabetic foot problems: prevention and management (NG19). 2015. https://www.nice.org.uk/guidance/ng19. Accessed 21 Feb 2023. 32045177

[104] Feng Y, Schlösser FJ, Sumpio BE (2011). The Semmes Weinstein monofilament examination is a significant predictor of the risk of foot ulceration and amputation in patients with diabetes mellitus. J Vasc Surg.

[105] Wang F, Zhang J, Yu J, Liu S, Zhang R, Ma X (2017). Diagnostic accuracy of monofilament tests for detecting diabetic peripheral neuropathy: a systematic review and meta-analysis. J Diabetes Res.

[106] McIllhatton A, Lanting S, Lambkin D, Leigh L, Casey S, Chuter V (2021). Reliability of recommended non-invasive chairside screening tests for diabetes-related peripheral neuropathy: a systematic review with meta-analyses. BMJ Open Diabetes Res Care.

[107] Dros J, Wewerinke A, Bindels PJ, van Weert HC (2009). Accuracy of monofilament testing to diagnose peripheral neuropathy: a systematic review. Ann Fam Med.

[108] Rayman G, Vas PR, Baker N, Taylor CG, Gooday C, Alder AI, Donohoe M (2011). The Ipswich touch test: a simple and novel method to identify inpatients with diabetes at risk of foot ulceration. Diabetes Care.

[109] Zhao N, Xu J, Zhou Q, Li X, Chen J, Zhou J (2021). Application of the Ipswich touch test for diabetic peripheral neuropathy screening: a systematic review and meta-analysis. BMJ Open.

[110] Hu A, Koh B, Teo MR (2020). A review of the current evidence on the sensitivity and specificity of the Ipswich touch test for the screening of loss of protective sensation in patients with diabetes mellitus. Diabetol Int.

[111] Kress S, Anderten H, Borck A, Freckmann G, Heinemann L, Holzmüller U (2021). Preulcerous risk situation in diabetic foot syndrome: proposal for a simple ulcer prevention score. J Diabetes Sci Technol.

[112] Cernea S, Raz I (2021). Management of diabetic neuropathy. Metabolism.

[113] Aszmann O, Tassler PL, Dellon AL (2004). Changing the natural history of diabetic neuropathy: incidence of ulcer/amputation in the contralateral limb of patients with a unilateral nerve decompression procedure. Ann Plast Surg.

[114] Partanen J, Niskanen L, Lehtinen J, Mervaala E, Siitonen O, Uusitupa M (1995). Natural history of peripheral neuropathy in patients with non-insulin-dependent diabetes mellitus. N Engl J Med.

[115] Tesfaye S, Vileikyte L, Rayman G, Sindrup SH, Perkins BA, Baconja M (2011). Painful diabetic peripheral neuropathy: consensus recommendations on diagnosis, assessment and management. Diabetes Metab Res Rev.

[116] Bondar A, Popa AR, Papanas N, Popoviciu M, Vesa CM, Sabau M (2021). Diabetic neuropathy: a narrative review of risk factors, classification, screening and current pathogenic treatment options (review). Exp Ther Med.

[117] Ziegler D, Tesfaye S, Spallone V, Gurieva I, Al Kaabi J, Mankovsky B (2022). Screening, diagnosis and management of diabetic sensorimotor polyneuropathy in clinical practice: international expert consensus recommendations. Diabetes Res Clin Pract.

[118] Price R, Smith D, Franklin G, Gronseth G, Pignone M, David WS, Armon C, Perkins BA, Bril V, Rae-Grant A, Halperin J, Licking N, O’Brien MD, Wessels SR, MacGregor LC, Fink K, Harkless LB, Colbert L, Callaghan BC (2022). Oral and topical treatment of painful diabetic polyneuropathy: practice guideline update summary: report of the AAN guideline subcommittee. Neurology.

[119] Gilron I, Bailey JM, Tu D, Holden RR, Weaver DF, Houlden RL (2005). Morphine, gabapentin, or their combination for neuropathic pain. N Engl J Med.

[120] Schlereth T (2020). Guideline, “diagnosis and non interventional therapy of neuropathic pain” of the German Society of Neurology (deutsche Gesellschaft für Neurologie). Neurol Res Pract.

[121] Finnerup NB, Attal N, Haroutounian S, McNicol E, Baron R, Dworkin RH, Gilron I, Haanpää M, Hansson P, Jensen TS, Kamerman PR, Lund K, Moore A, Raja SN, Rice AS, Rowbotham M, Sena E, Siddall P, Smith BH, Wallace M (2015). Pharmacotherapy for neuropathic pain in adults: a systematic review and meta-analysis. Lancet Neurol.

[122] Juhn MS, Parsons B, Varvara R, Sadosky A (2015). Pregabalin for painful diabetic peripheral neuropathy: strategies for dosing, monotherapy vs. combination therapy, treatment-refractory patients, and adverse events. Curr Med Res Opin.

[123] Balanaser M, Carley M, Baron R, Finnerup NB, Moore RA, Rowbotham MC, Chaparro LE, Gilron I (2023). Combination pharmacotherapy for the treatment of neuropathic pain in adults: systematic review and meta-analysis. Pain.

[124] Tesfaye S, Sloan G, Petrie J, White D, Bradburn M, Julious S, Rajbhandari S, Sharma S, Rayman G, Gouni R, Alam U, Cooper C, Loban A, Sutherland K, Glover R, Waterhouse S, Turton E, Horspool M, Gandhi R, Maguire D, Jude EB, Ahmed SH, Vas P, Hariman C, McDougall C, Devers M, Tsatlidis V, Johnson M, Rice ASC, Bouhassira D, Bennett DL, Selvarajah D, OPTION-DM trial group (2022). Comparison of amitriptyline supplemented with pregabalin, pregabalin supplemented with amitriptyline, and duloxetine supplemented with pregabalin for the treatment of diabetic peripheral neuropathic pain (OPTION-DM): a multicentre, double-blind, randomised crossover trial. Lancet.

[125] Duarte RV, Nevitt S, Maden M, Meier K, Taylor RS, Eldabe S, de Vos CC (2021). Spinal cord stimulation for the management of painful diabetic neuropathy: a systematic review and meta-analysis of individual patient and aggregate data. Pain.

[126] Strand NH, Burkey AR (2022). Neuromodulation in the treatment of painful diabetic neuropathy: a review of evidence for spinal cord stimulation. J Diabetes Sci Technol.

[127] Petersen EA, Stauss TG, Scowcroft JA, Brooks ES, White JL, Sills SM, Amirdelfan K, Guirguis MN, Xu J, Yu C, Nairizi A, Patterson DG, Tsoulfas KC, Creamer MJ, Galan V, Bundschu RH, Mehta ND, Sayed D, Lad SP, DiBenedetto DJ, Sethi KA, Goree JH, Bennett MT, Harrison NJ, Israel AF, Chang P, Wu PW, Argoff CE, Nasr CE, Taylor RS, Caraway DL, Mekhail NA (2022). High-frequency 10-kHZ spinal cord stimulation improves health-related quality of life in patients with refractory painful diabetic neuropathy: 12-month results from a randomized controlled trial. Mayo Clin Proc Innov Qual Outcomes.

[128] Raghu ALB, Parker T, Aziz TZ, Green AL, Hadjipavlou G, Rea R, FitzGerald JJ (2021). Invasive electrical neuromodulation for the treatment of painful diabetic neuropathy: systematic review and meta-analysis. Neuromodulation.

[129] Zeng H, Pacheco-Barrios K, Cao Y, Li Y, Zhang J, Yang C, Fregni F (2020). Non-invasive neuromodulation effects on painful diabetic peripheral neuropathy: a systematic review and meta-analysis. Sci Rep.

[130] Binns-Hall O, Selvarajah D, Sanger D, Walker J, Scott A, Tesfaye S (2018). One-stop microvascular screening service: an effective model for the early detection of diabetic peripheral neuropathy and the high-risk foot. Diabet Med.

[131] Hussain MA, Al-Omran M, Salata K, Sivaswamy A, Verma S, Forbes TL (2019). A call for integrated foot care and amputation prevention pathways for patients with diabetes and peripheral arterial disease across Canada. Can J Public Health.

[132] Selvarajah D, Kar D, Khunti K, Davies MJ, Scott AR, Walker J, Tesfaye S (2019). Diabetic peripheral neuropathy: advances in diagnosis and strategies for screening and early intervention. Lancet Diabetes Endocrinol.

[133] Lewis JE, Morris K, Powell T, Thomas RL, Owens DR (2020). Combining diabetic foot and retinopathy screening: a step in the right direction?—a feasibility study. SAGE Open Med.

[134] Carmichael J, Fadavi H, Ishibashi F, Howard S, Boulton AJM, Shore AC, Tavakoli M (2022). Implementation of corneal confocal microscopy for screening and early detection of diabetic neuropathy in primary care alongside retinopathy screening: results from a feasibility study. Front Endocrinol.

[135] Formosa C, Gatt A, Chockalingham N (2016). A critical evaluation of existing diabetic foot screening guidelines. Rev Diabet Stud.

[136] Pérez-Panero AJ, Ruiz-Muñoz M, Cuesta-Vargas AI, Gónzalez-Sánchez M (2019). Prevention, assessment, diagnosis and management of diabetic foot based clinical practice guidelines: a systematic review. Medicine.

[137] Tan MKH, Goodall R, Hughes W, Langridge B, Shalhoub J, Davies AH (2020). A methodological assessment of diabetic foot syndrome clinical practice guidelines. Eur J Vasc Endovasc Surg.

